# Network Pharmacology and Molecular Docking Analysis of *Morinda citrifolia* Fruit Metabolites Suggest Anxiety Modulation through Glutamatergic Pathways

**DOI:** 10.3390/life14091182

**Published:** 2024-09-19

**Authors:** Zaina Allyson A. Rivera, Nicholas Dale D. Talubo, Heherson S. Cabrera

**Affiliations:** 1 School of Chemical, Biological and Materials Engineering and Sciences, Mapúa University, Manila 1002, Philippines; zaarivera@mymail.mapua.edu.ph (Z.A.A.R.); nddtalubo@mymail.mapua.edu.ph (N.D.D.T.); 2 School of Graduate Studies, Mapúa University, Manila 1002, Philippines; 3 Department of Biology, School of Health Sciences, Mapúa University, Makati 1200, Philippines

**Keywords:** *Morinda citrifolia*, noni fruit, anxiety, nonioside, in silico analysis

## Abstract

The fruit of *Morinda citrifolia*, also known as the noni tree, has been extensively used in Polynesian culture as an alternative medicine to various diseases. Recent studies have pointed out its anxiolytic activity in vitro and in mouse models. Despite the effectiveness of developed anxiolytic drugs in the market, the potential side effects of these medications have led people to resort to traditional medicine such as *M. citrifolia.* However, evidence regarding its anti-anxiety characteristics is still lacking to this day. Hence, this preliminary study implemented combined network pharmacology and molecular docking to validate its anti-anxiety claims. This study highlighted the bioactive compounds of the *M. citrifolia* fruit part to have excellent absorption, distribution, metabolism, excretion, and toxicity (ADMET) properties, particularly their outstanding oral bioavailability and blood–brain barrier penetration, both of which are essential considerations to ensure the effectiveness of anxiolytic drugs to arrive at the site of action. Moreover, noni fruit metabolites target genes involved in glutamatergic synapse pathways, which have been significantly associated with anxiety. Through molecular docking, selected compounds exhibited a strong binding affinity towards GRIA2 and PRKCA, both of which have connections with glutamatergic pathways. With all things considered, the results established that the noni fruit potentially contains therapeutic agents that elicit anti-anxiety potential. Through this, the promotion of a more sustainable, accessible, and affordable treatment of anxiety could be developed.

## 1. Introduction

The overall quality of life across the world has been steadily increasing in the 20th and 21st centuries. Despite this, the incidence of anxiety has continued to plague those afflicted across socio-economic statuses and geographical realities. In fact, anxiety is known as one of the most common psychiatric disorders, with it being more common in women than in men, with a 2:1 ratio [1]. It is estimated that anxiety and related disorders cost at least USD 6.3 trillion globally and are exacerbated by the unequal access to proper treatment and resources in affected population groups [2].

The steep cost of anxiety in health and the economy has prompted researchers to examine this phenomenon from multiple angles. An accepted definition is that anxiety is a fear-based state or a response to a perceived threatening event, either in the future or the past [1]. While worrying is known to be an ultimately “human” emotion, in excess, anxiety has been described as pathological and potentially debilitating, with the fifth version of the Diagnostic and Statistical Manual of Mental Illnesses (DSM-5) defining it as a Generalized Anxiety Disorder (GAD) if criteria like difficulty to control excessive worrying, a high-frequency occurrence for the last six months, and physiological symptoms like increased fatigue and muscle tension are present [3]. Beyond this, studies on the pathophysiology of anxiety have been undertaken, with the central nervous system (CNS) being implicated in its overall phenomenon, particularly the amygdala, prefrontal cortex, and hippocampus [4].

The research into anxiety has been translated into treatment paradigms of various levels of congruence. Mental health practitioners tackle anxiety both physically and mentally. Mentally, anxiety is tackled through psychotherapy with techniques such as Cognitive Behavioral Therapy (CBT) and Acceptance and Commitment Therapy [5]. The effectiveness of such techniques has been demonstrated by reduced symptom severity and reduced remission rate [6]. Physically, anxiety can be tackled with the use of pharmaceuticals. Overall, various pharmacotherapy drugs have been developed for anxiety or other disorders whose primary symptom is anxiety. These include alpha- and beta-adrenergic medications, antihistamines, antipsychotics, azapirones, GABAergic medications, mixed antidepressants, serotonin-norepinephrine reuptake inhibitors (SNRIs), and selective serotonin reuptake inhibitors (SSRIs) [7]. These drugs have undergone regulatory testing and have proven their effectiveness in patients. However, there is still a prevalent fear of side effects from taking such medications [7].

The potential side effects of pharmacotherapy have prompted interest in alternative substances that can produce the same effects. Culturally, this renewed the relevance of traditional medicinal systems like Ayurvedic medicine traditions and traditional Chinese medicines (TCM) [8]. In science, this interest has served to encourage scientists to improve the pre-existing pharmaceuticals or hunt for new substances artificially or in the natural world. This has led to studies on popular herbs, such as ashwagandha (*Withania somnifera*), which is known to affect anxiety when used [9]. One such herb is *Morinda citrifolia*, or the noni plant, which is known to be a fruit-bearing tree existing across Southeast Asia, Australasia, and various islands in the Pacific [10]. It has been used in traditional Polynesian medicine and has been experimentally studied for anxiolytic effects [11,12].

In this study, the fruit of interest, *M. citrifolia*, is examined through network pharmacology. Various compounds of the fruit were screened to determine whether compounds from *M. citrifolia* exhibit anti-anxiety properties and to investigate their ability to cross the blood–brain barrier alongside other absorption, distribution, metabolism, excretion, and toxicity (ADMET) properties. Additionally, the aim was to identify compounds with high potential to target anxiety-related proteins through molecular docking analysis. The results of this study should guide further therapeutic considerations for the noni fruit.

## 2. Materials and Methods

### 2.1. Screening of Morinda citrifolia Fruit Compounds

To ensure that only metabolites of the fruit part are obtained, the Indian Medicinal Plants, Phytochemistry And Therapeutics 2.0 (IMPPAT) online database (https://cb.imsc.res.in/imppat, accessed on 12 June 2024) was utilized [13,14]. Similarly, additional compounds were searched through multiple pieces of literature [11,15,16,17,18]. The compounds were screened using ADMETLab3.0 (https://admetmesh.scbdd.com, accessed on 16 June 2024) [19] under the Lipinski rule, Pfizer rule, blood–brain barrier (BBB) penetration, and human oral bioavailability 20% (F20%). A compound was accepted under the Lipinski rule if it had no more than one violation among the following properties: molecular weight (MW ≤ 500), logarithm of the n-octanol/water distribution coefficient (logP ≤ 5), hydrogen acceptor (Hacc ≤ 10), and hydrogen donor (Hdon ≤ 5). A compound was accepted according to the Pfizer rule if it did not satisfy the following properties: high logP (>3) and low topological polar surface area (TPSA < 75). For BBB permeability and F20%, compounds with a score of 0 to 0.3 (≤0.3) were deemed excellent and were retained.

### 2.2. Prediction of Anxiety-Related Targets of M. citrifolia Fruit

The compound targets were acquired by using two databases: SwissTargetPrediction (http://www.swisstargetprediction.ch, accessed on 17 June 2024) [20] and SuperPred (https://prediction.charite.de, accessed on 17 June 2024) [21]. For the former, the cut-off probability was set to 0.7 (≥0.7), while for the latter, the probability and model accuracy were set to 80% (≥80%). On the other hand, GeneCards (https://www.genecards.org, accessed on 7 June 2024) was used to search for anxiety-related genes [22]. Only the top 25% of presented genes were acquired. Finally, the intersected genes of the compound targets and anxiety-related genes were derived using Venny 2.1 (https://bioinfogp.cnb.csic.es/tools/venny/, accessed on 30 June 2024) [23] to identify the anxiety-related targets of *M. citrifolia* fruit compounds.

### 2.3. Network Construction

A compound–target network was constructed using the Pandas Library and NetworkX Library (Python ver. 3.12.14), where the top 10 compounds were identified by degree. The common anxiety-related targets previously determined were used to construct the protein–protein interaction (PPI) network in the STRING database (https://string-db.org, accessed on 15 July 2024) [24]. The proteins were restricted to *Homo sapiens* only. The PPI network was further refined using Cytoscape (version 3.10.0) [24]. Moreover, the CytoHubba extension was used to identify the top 10 hub genes by degree [25]. Lastly, the compound–pathway interaction was created using ggplot2 and ggalluvial in R (ver. 4.4.1) and MyGene.info (https://mygene.info, accessed on 23 July 2024) to query the KEGG membership.

### 2.4. Enrichment Analysis

The gene ontology (GO), Kyoto Encyclopedia of Genes and Genomes (KEGG) pathway, Reactome pathway, and DisGeNET enrichment analysis were performed in the Database for Annotation, Visualization, and Integrated Discovery (DAVID) (https://david.ncifcrf.gov, accessed on 30 June 2024) [26,27]. Gene restrictions were set on *Homo sapiens*. The website SRplot was then used for visualization (https://www.bioinformatics.com.cn/, accessed on 3 July 2024) [28].

### 2.5. Molecular Docking

Blind docking was executed using CB-Dock2 (https://cadd.labshare.cn/cb-dock2/index.php, accessed on 28 July 2024) [29,30] to validate the interaction between selected top compounds of *M. citrifolia* fruit, controls, and anxiety key targets, glutamate ionotropic receptor AMPA type subunit 2 (GRIA2) and protein kinase c alpha (PRKCA). The .sdf files of the compounds were retrieved from PubChem [31]. The controls used were known compounds that bind to GRIA2 or PRKCA which were Staurosporine [32], 14-(R)-Hydroxy-retro-vitamin A [33], and Parampanel [34]. The protein structures of GRIA2 (ID: 7F3O) and PRKCA (ID: 4DNL) were retrieved from the RCSB Protein Data Bank (PDB) (https://www.rcsb.org, accessed on 28 July 2024). Before docking, the proteins were prepared by manually removing unnecessary water, ligand groups, and heteroatoms and adding polar hydrogen using Discovery Studio Visualizer 2021. The ligand–protein complexes from the same cavity were downloaded, and 2D and 3D diagrams were generated through Discovery Studio Visualizer.

## 3. Results

### 3.1. Bioactive Compounds of Morinda citrifolia Fruit

The literature and IMPPAT search revealed 111 compounds identified in the noni fruit. These compounds were then filtered using selected ADMET criteria to retain only the compounds that possess drug-like properties. Exceptions were made for some nonioside compounds that met all criteria except the Lipinski rule since noniosides are unique compounds existing only in *M. citrifolia*. Table 1 presents the screening scores of these compounds. After filtering, about 31 compounds remained, including nonioside A, D, and E.

### 3.2. Identification of Anxiety-Related Targets of M. citrifolia Fruit

**Figure 1:**
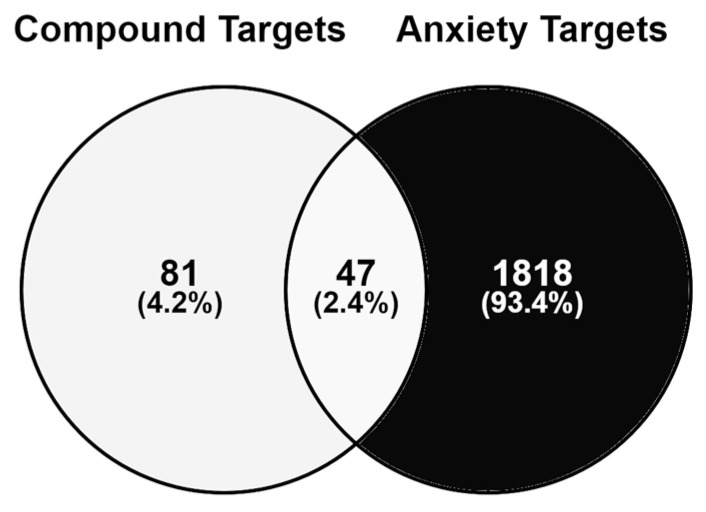
The intersecting targets between *Morinda citrifolia* and anxiety using a Venn diagram.

The protein targets of the 31 bioactive compounds were identified using two databases to ensure comprehensive coverage. Initially, 438 compound targets were identified, but after removing duplicates, only 128 unique targets remained. To focus on anxiety-related targets of *M. citrifolia*, a Venn diagram was created to compare the 128 compound targets with 1865 anxiety genes previously obtained from GeneCards (Figure 1). The intersection revealed 47 targets, indicating that 47 out of the 128 targets of *M. citrifolia* compounds were anxiety related. This implies the potential of noni fruit to treat anxiety, given its substantial number of anxiety-related targets.

### 3.3. Identification of Key Compounds and Hub Genes

**Figure 2:**
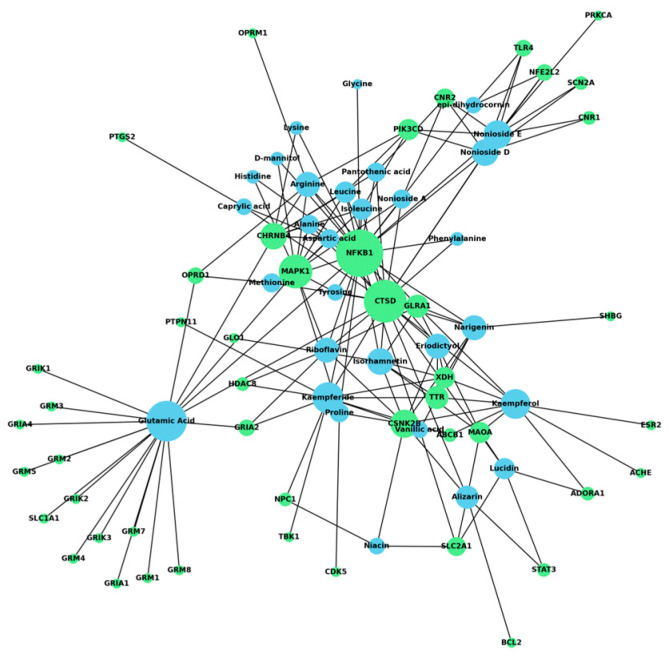
Compound–target network of *Morinda citrifolia* fruit parts and anxiety-related targets. Blue circles represent the compounds, while green circles represent the targets. The size of each circle varies depending on the degree of connection, with larger circles indicating more interactions.

**Figure 3:**
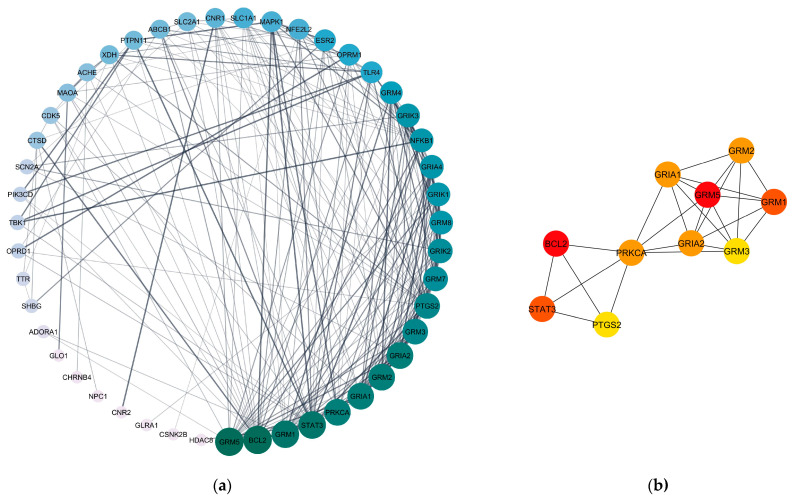
Protein–protein interaction network of *M. citrifolia* fruit anxiety gene targets (**a**) protein–protein interaction of the 47 anxiety gene targets. (**b**) the interaction of the top 10 hub genes by degree. The darker the color of the nodes, the more interactions.

A compound–target network was constructed to identify the key compounds of the noni fruit (Figure 2). Here, only the 47 anxiety-related protein targets were included. The network revealed that only 30 out of 31 compounds target anxiety protein targets, with pyridoxine having no anxiety-related targets. Furthermore, the top 10 compounds by degree were glutamic acid, kaempferide, kaempferol, nonioside E, isorhamnetin, nonioside D, arginine, eriodictyol, narigenin, and riboflavin. These compounds were considered essential compounds due to their relatively greater number of target interactions. Meanwhile, the protein–protein interaction network illustrated in Figure 3a was the basis for identifying the hub genes. As shown in Figure 3b, the top 10 hub genes by degree were the following: GRM5, BCL2, STAT3, GRM1, GRIA2, GRM2, PRKCA, GRIA1, GRM3, and PTGS2. These proteins signify the ten proteins that interact with many other proteins; hence, they play a massive role in the regulation of the network as a whole.

### 3.4. Gene Ontology and Pathway Enrichment Analysis

**Figure 4:**
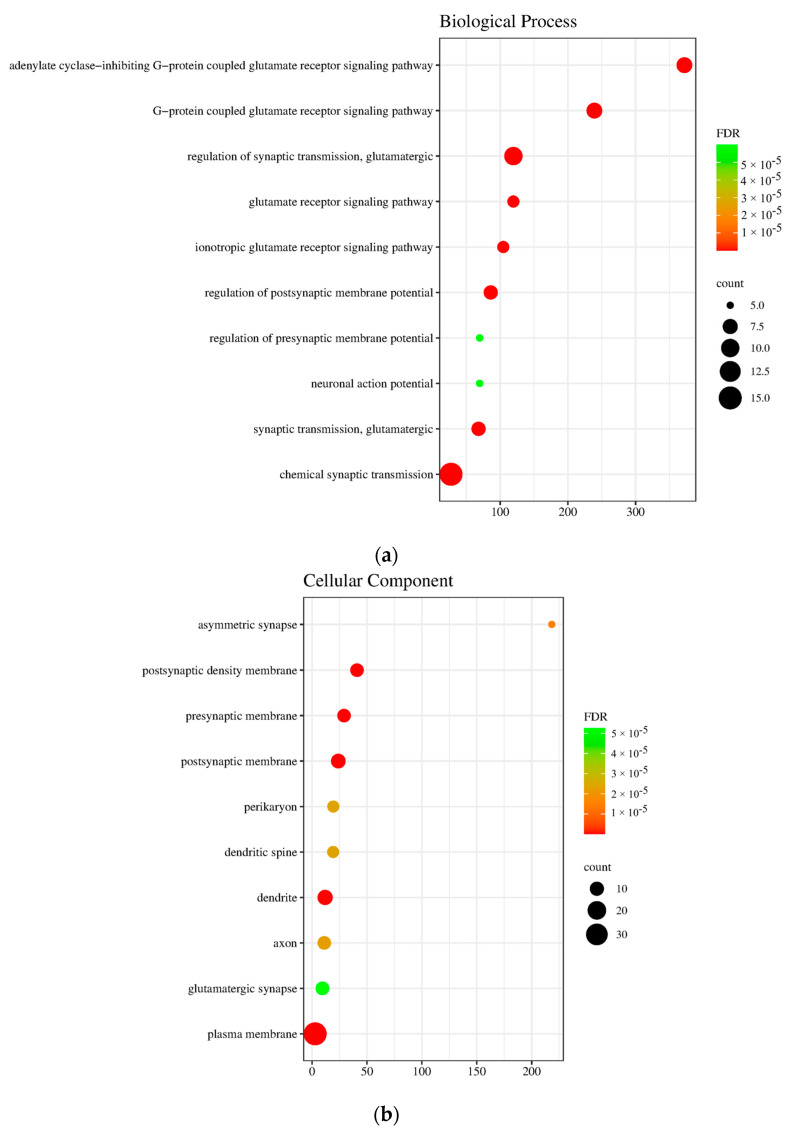

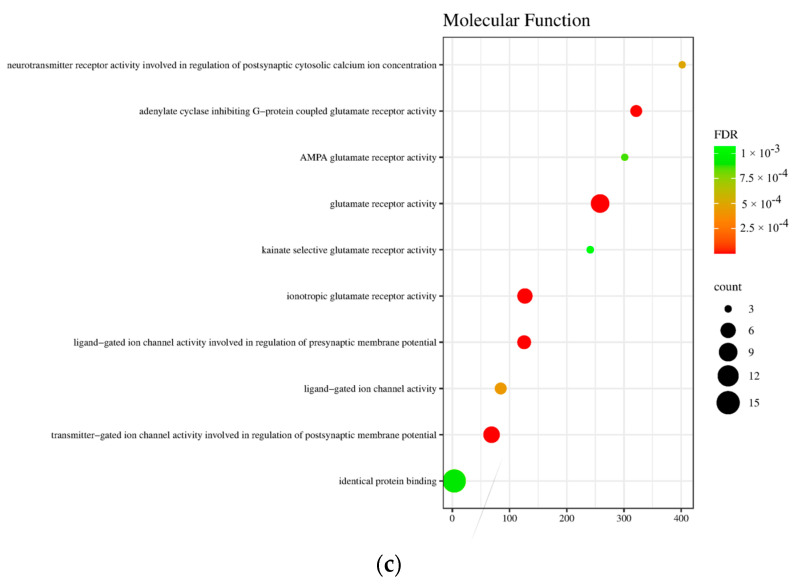
Gene ontology enrichment analysis of *Morinda. citrifolia* anxiety-related protein targets: (**a**) biological process, (**b**) cellular component, (**c**) molecular function. Ranked by fold enrichment (x-component).

The gene ontology (GO) enrichment analysis of the 47 common targets was summarized in Figure 4, showing the top 10 ranked by fold enrichment with the lowest FDR values. The biological processes indicate that the targets are primarily involved in glutamate receptor signaling pathways. Cellular component analysis, on the other hand, revealed their association with synapses and synaptic membranes, implying that the targets work around the neurons. These results were further supplemented by the molecular function that generally highlights neurotransmitter receptor and glutamate receptor activity.

**Figure 5:**
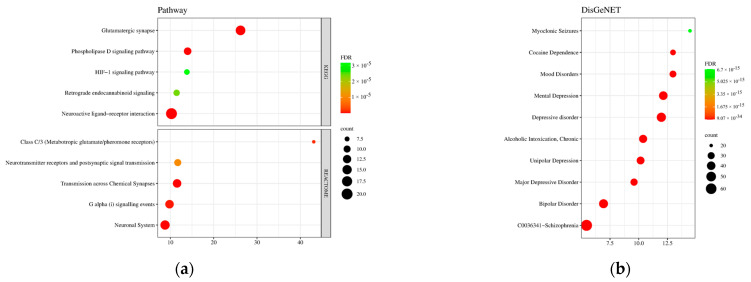
Pathway and disease enrichment analysis of *M. citrifolia* anxiety-related targets. (**a**) KEGG and reactome pathway analysis; (**b**) DisGeNET disease analysis.

Interestingly, the findings in GO enrichment analysis were further supported by the KEGG and Reactome pathway enrichment analysis (Figure 5a). Here, only the top five lowest FDR values were taken and were ranked according to fold enrichment. The critical pathways involved were the glutamatergic synapse and G-protein-coupled receptors (GPCR) glutamate/pheromone signaling, along with neurotransmitter receptors, neuroactive ligand–receptor interactions, and generally neuronal and synaptic activity pathways. Evidently, the targets have a huge role in synaptic transmission, signal transduction, cellular responses, and neuronal systems. The overall findings in GO and pathway enrichment analysis suggest that *M. citrifolia* fruit bioactive compounds can regulate these processes and pathways due to their ability to target diverse proteins involved.

DisGeNET results also presented relevant diseases associated with the common targets (Figure 5b). The top four were myoclonic seizures, cocaine dependence, mood disorders, and depression. Most involve neurological and psychiatric conditions, suggesting the protein targets’ broad impact on brain function. Moreover, the diseases and disorders appear to have links with anxiety or exhibit anxiety-like symptoms.

### 3.5. Identification of Compound–Pathway Interactions

**Figure 6:**
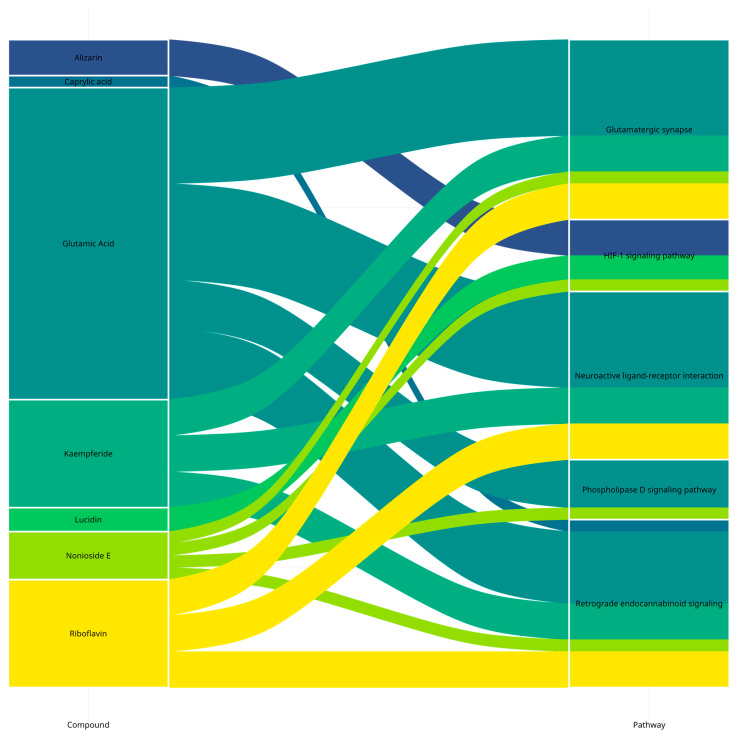
*Morinda citrifolia* major compound–KEGG pathway interactions.

An alluvial plot was constructed based on the results of the KEGG pathway analysis to illustrate a representation of how *M. citrifolia* compounds, their top 10 anxiety-related hub gene targets, and pathways interact (Figure 6). The flows linking the compound and pathway denote its interaction, suggesting that the compound targets a gene involved in the pathway. The thickness of each flow correlates to the number of hub genes the compound targets. The thicker the flow, the more genes associated with the pathway the compound targets. Of the seven compounds, four compounds were previously identified key compounds, namely glutamic acid, kaempferide, nonioside E, and riboflavin. These four compounds target several genes in the glutamatergic synapse pathway. Other significant pathways that have many target genes of the four key compounds involved are the neuroactive ligand–receptor interaction and retrograde endocannabinoid signaling pathway. This network suggests the possible synergistic impact of noni fruit compounds against anxiety through key pathways that influence the disorder.

### 3.6. Molecular Docking

Based on the networks constructed, glutamic acid targets five out of the ten hub genes. GRIA2 shows multiple predicted interactions with the compounds, including glutamic acid, kaempferide, and riboflavin. Of the two noniosides, only nonioside E targets a hub gene, PRKCA. With this rationale, these compounds and targets were the only ones used for molecular docking. Blind docking was performed to simulate the potential interactions of the selected compounds and protein targets. The Vina scores for the same cavity were obtained for the docking of GRIA2 with glutamic acid, kaempferide, and riboflavin. Additionally, perampanel was used as the control. As observed in Table 2, all compounds, including the control, spontaneously bind with GRIA2 in the same cavity. This result indicates the successful binding of the compounds with GRIA2 and the potential cooperative stimulating effect on the protein.

**Figure 7:**
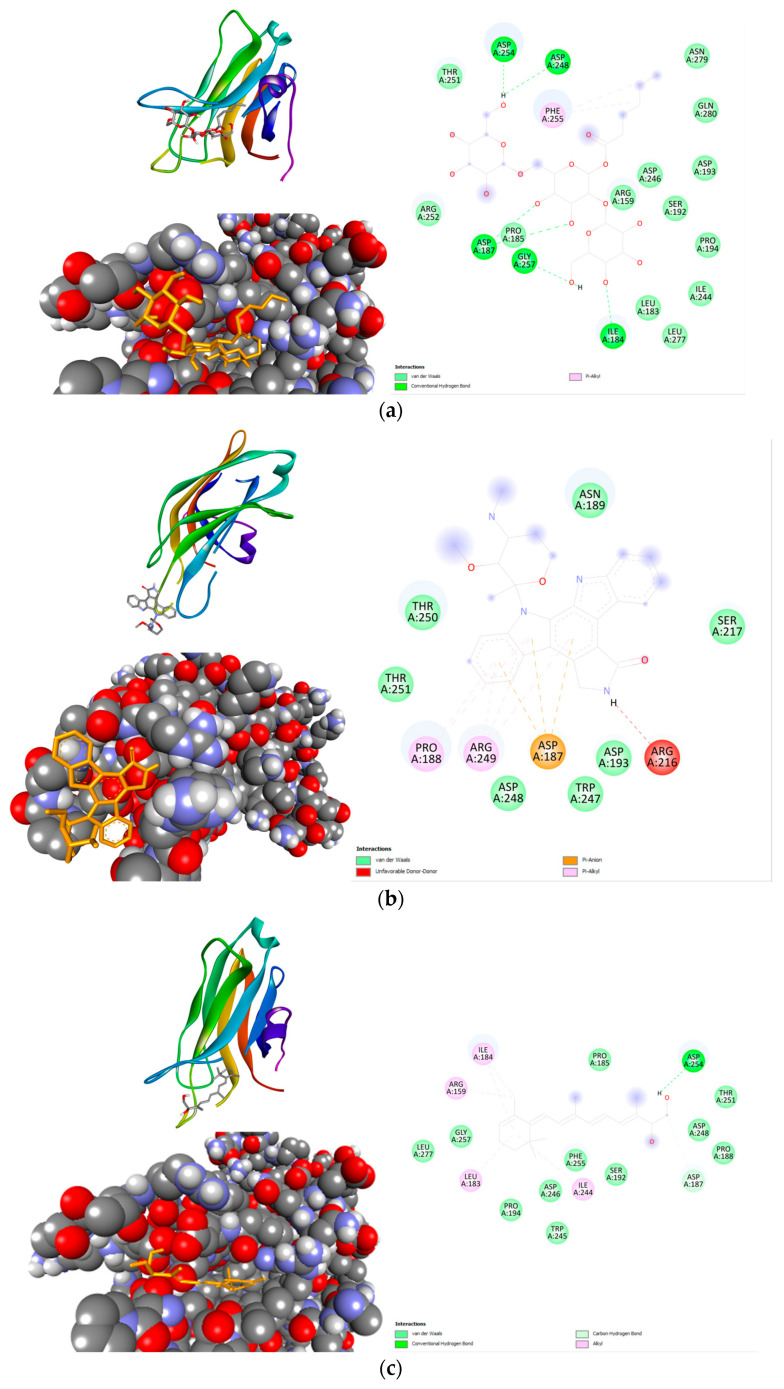
Compound–protein docking shown in 2D and 3D diagrams. (**a**) Nonioside E-PRKCA complex, (**b**) Staurosporine-PRKCA complex, (**c**) 14-(R)-Hydroxy-retro-vitamin A-PRKCA complex.

Moreover, nonioside E was docked with PRKCA to explore the impact of the unique compound on anxiety. The results in Table 3 suggest that nonioside E favorably binds with PRKCA and shows stronger binding affinity compared to the controls. This is further supported by Figure 7, which illustrates that nonioside E (Figure 7a) forms more conventional hydrogen bond interactions with the protein than the controls (Figure 7b,c).

## 4. Discussion

The use of natural compounds like phytochemicals and mycochemicals has been well-established in the popular and scientific literature [35]. There is still, however, a need for more clarity between the effectiveness of these claims and what can be scientifically measured. In this paper, the compounds from *M. citrifolia* with high potential to target anxiety-related proteins were identified and analyzed through network pharmacology and molecular docking. It was discovered that noni fruit contains diverse bioactive compounds that target anxiety-related proteins, supporting its anti-anxiety claims. Interestingly, among these compounds are the noniosides, which are trisaccharide fatty acid esters that can be uniquely isolated specifically from the noni fruit [36]. Moreover, these compounds were predicted to have good ADMET properties, specifically their ability to cross the blood–brain barrier (BBB) and their oral bioavailability. Overall, the bioactive compounds of the noni fruit were shown to have the potential to target processes and pathways with a strong relationship to anxiety- or fear-based responses like the glutamatergic and neuronal systems. The two proteins selected for further analysis, GRIA2 and PRKCA, are involved in the glutamatergic synapse and were found to have good binding activity with the selected compounds, suggesting that the compounds could potentially influence the proteins.

As stated earlier, the compounds of the noni fruit were found to number at least 31, which passed the set thresholds for the Lipinski rule, Pfizer rule, blood–brain barrier (BBB) penetration, and human oral bioavailability. As of 2024, the complete phytochemical profile of the noni tree has yet to be accurately compiled, and currently, different compounds are still being reported by different studies. Nevertheless, there is still some agreement on the potential compounds present in the fruit. First, flavonoids, tannins, and steroids were found in the fruit juice and were noted to have antifungal activity against *C. albicans* and *C. krusei* [37]. Furthermore, secondary metabolites such as polyphenols, alkaloids, and glycosides were also identified to be present, and the antioxidant and antimicrobial activity of the juice has been proven in vitro [17]. Unique compounds like the noniosides were identified, with at least eight varieties found [36]. The exact effects of these compounds are still unclear as they are implicated along with other bioactive compounds in identification studies. However, some, like nonioside A, have been noted to be anti-inflammatory [18]. Despite the difficulty in isolating a specific compound to an effect, the potential impact of the fruit juice can still be studied. In particular, as an anxiolytic, the noni fruit has been found to display binding inhibition to a GABA_A_ receptor in an innovative GABA_A_ receptor-binding assay, indicating its anti-anxiety effects [38]. Interestingly, these findings were replicated with behavioral and mechanistic studies on mice where the results indicated the involvement of the benzodiazepine-GABA_A_ergic and serotonergic receptors for anti-anxiety and the serotonergic and noradrenergic systems for anti-depression [39]. These findings provide reasonable evidence that *M. citrifolia*, particularly the metabolites of its fruit, exhibit anti-anxiety activity. This suggests its potential to be an alternative form of treatment, promoting accessibility, affordability, and sustainability in healthcare.

Currently, various drugs can be used for the treatment of anxiety [7]. One of the most common options are benzodiazepines. These classes of drugs are known to target GABA_A_ receptors to reduce anxiety and have been found to share transmembrane-binding sites with intravenous anesthetics and inhibitory modulators [40]. However, these drugs are known to be potentially addictive, requiring control over their distribution [41]. Other popular options are reuptake inhibitors for serotonin and norepinephrine. Unlike benzodiazepines, SSRIs and SNRIs are designed to bind to serotonin or norepinephrine transporters to inhibit anxiety or depression [42]. Notably, the two drugs offer unique pathways for managing anxiety. However, a common denominator between the two is their capability to cross the blood–brain barrier. Hence, a characteristic needed for lead compounds is their ability to cross the BBB. Additionally, since the noni fruit is usually consumed orally, the metabolites should reach the systemic circulation. These properties are ensured by integrating BBB penetration and oral bioavailability as the criteria for ADMET screening. Aside from these, Lipinski and Pfizer’s rules were also employed as criteria to consider the drug-likeliness of the compounds. Since noniosides were unique to the noni fruit, it was decided to retain these compounds even if they violated the Lipinski rule of five. While the Lipinski rule of five is a prominent rule of thumb in screening, the most favorable exceptions to the rule are natural products and that drugs can still exhibit good activity even if they do not conform to the rules [43]. Nonetheless, in this study, all identified 31 bioactive compounds present excellent BBB penetration and oral bioavailability, suggesting these metabolites have the potential to effectively reach the site of action, such as the GABA_A_ergic and serotonergic receptors, which have been previously noted to be regulated by the noni fruit. However, for this study, focus on the glutamatergic pathway was given. An emerging novel treatment option for anxiety disorders, ketamine, is also known to target this pathway [44].

The ubiquitousness of anxiety in the human psyche gives it a premier role in both its normal and diseased functioning. As such, anxiety is implicated in multiple types of disorders, including psychosis and mood disorders [45,46]. The ability to manage such disorders is crucial, and anxiety may be a key piece in their treatment. In fact, the target genes of this paper appear consistent with known genes involved in various psychiatric disorders such as schizophrenia and mood disorders. The target genes were also implicated in the glutamatergic synapse pathway. Glutamate is known to be released during stress and has been proven to produce anxiety-like behavior in animals [47]. Glutamate receptors can enhance anxiety, and the blocking of which has produced anxiolytic effects on testing [48]. Furthermore, the balance of Glutamate-GABA is implicated in anxiety disorders [48]. These findings support the results of this study since overlapping genes exist between the KEGG term neuroactive ligand–receptor interaction, which contains interactions with GABA and serotonin neurotransmitters, and the glutamatergic synapse pathway. Other pathways implicated in the genes are involved with anxiety in different ways. First, the phospholipase D signaling pathway was discovered to have interactions with metabotropic glutamate receptors (mGluRs) and has downstream effects on fear memory encoding and anxiety [49]. Second, hypoxia induces the involvement of hypoxia-inducible factors (HIFs), and severe cases do contribute to the development of anxiety and depression [50]. Potentially, the identified metabolites of noni fruit target critical proteins involved in these pathways, thereby possibly regulating anxiety.

The proteins retrieved for molecular docking are involved in the glutamatergic pathway. In this paper, blind docking was used to computationally evaluate the interactions of the chosen compounds and the target genes. Blind docking is a strategy where both the binding poses and the binding regions of the protein are determined [29]. Depending on the settings, blind docking could be very advantageous since the identification of the binding region is unbiased and can be iterated across the entire protein [51]. Principally, with blind docking, the potential protein–ligand complex can be simulated; however, a hefty computational cost is incurred depending on the size of the protein and the number of binding regions. The favorable conformation is determined using the Vina score, which is a weighted sum of intermolecular and intramolecular interactions with energy terms, such as Van der Waals, coulombic, and desolvation terms, combined [52]. Both intermolecular and intramolecular interactions contribute to a lower Vina score, which indicates a higher binding efficiency. One notable interaction is with nonioside E, which bound to PRKCA well due to hydrogen bonds. The amino acid residues were also examined for all compounds. For PRCKA, it was found that none of the compounds directly interact with Thr497, Thr638, or Ser657, which are the key phosphorylation sites for PKC-α activation [53]. However, they interact with nearby residues like THR251 and ASP254, which may influence the overall structure and stability of PKC-α. For GRIA2, the examined compounds do not appear to directly interact with the key Q607, G609, or D611 residues. However, the general interaction with other residues in the binding pocket could still influence GluA2′s function because the nearby residues affect the conformational stability of GluA2 and its rectification properties, especially if they impact nearby regions like the M2 domain [54].

As an in silico study, the dynamics of the effect of the noni fruit juice on anxiety can be explored. However, the results of the study are limited by the current state of the databases and software use. While the relationship of the compounds of the noni fruit juice to the glutamatergic and GABA_A_ergic pathways were found, to truly evaluate its effects, in vitro studies on the receptors of interest must be conducted to analyze the exact effects of the juice. Furthermore, the intake of noni fruit juice may need to be experimentally evaluated in terms of its ability to reach the brain. Behavioral studies on rats can become a valuable tool to evaluate both its effectiveness in reducing anxiety and the ability of the compounds to cross the blood–brain barrier. This study can pave the way for the identification of the exact mechanisms and receptors for further experimental study. However, efforts must be made to improve the data on the noni fruit juice by determining the exact makeup of compounds. Furthermore, the various noniosides isolated must be studied individually for their effects and characteristics to discover the unique contribution that noni fruit juice can give or the various diseases it can remedy.

## 5. Conclusions

This study integrated network pharmacology and molecular docking to evaluate the potency of *M. citrifolia* fruit compounds on anxiety. Findings revealed that the noni fruit contains metabolites that exhibit outstanding ADMET properties, mainly being able to cross the blood–brain barrier and excellent oral bioavailability. Moreover, these bioactive compounds are likely to regulate anxiety-related protein targets that play significant roles in the glutamatergic synapse pathway and neuroactive ligand–receptor interaction pathway. Molecular docking results also highlighted strong protein–ligand interactions between GRIA2 and PRKCA and selected compounds. Notably, nonioside E, a compound unique to noni fruit, presented a favorable binding affinity towards PRKCA, suggesting its compelling therapeutic potential. Convincingly, this study explored the potential of noni fruit to treat anxiety and provided possible lead compounds from the fruit. Further investigation into the effects of the noni fruit in vitro and in vivo is recommended to develop a more thorough understanding of its therapeutic effect. Additionally, studies focusing on the bioactive compounds of *M. citrifolia*, specifically noniosides, could be a promising path to explore.

## Figures and Tables

**Table 1:** Screening criteria of *M. citrifolia* fruit compounds.

No	Compound Name	CID	Molecular Weight	Lipinski	Pfizer	BBB	f20
1	Alanine	5950	89.09	Passed	Passed	0.0182	0.0013
2	Alizarin	6293	240.21	Passed	Passed	0.0108	0.0340
3	Arginine	6322	174.2	Passed	Passed	0.0081	0.0003
4	Aspartic acid	5960	133.1	Passed	Passed	0.1476	0.0073
5	Caprylic acid	379	144.21	Passed	Passed	0.1854	0.2702
6	D-mannitol	6251	182.17	Passed	Passed	0.0326	0.1245
7	epi-dihydrocornin	44583983	390.4	Passed	Passed	0.1004	0.1847
8	Eriodictyol	440735	288.25	Passed	Passed	0.0008	0.0900
9	Glutamic Acid	33032	147.13	Passed	Passed	0.0790	0.0013
10	Glycine	750	75.07	Passed	Passed	0.1859	0.0006
11	Histidine	6274	155.15	Passed	Passed	0.0480	0.0002
12	Isoleucine	6306	131.17	Passed	Passed	0.0720	0.0103
13	Isorhamnetin	5281654	316.26	Passed	Passed	0.0001	0.0499
14	Kaempferide	5281666	300.26	Passed	Passed	0.0019	0.0970
15	Kaempferol	5280863	286.24	Passed	Passed	0.0010	0.0845
16	Leucine	6106	131.17	Passed	Passed	0.0056	0.0037
17	Lucidin	10163	270.24	Passed	Passed	0.0030	0.0069
18	Lysine	5962	146.19	Passed	Passed	0.0126	0.0017
19	Methionine	6137	149.21	Passed	Passed	0.0054	0.0005
20	Narigenin	439246	272.25	Passed	Passed	0.0002	0.0020
21	Niacin	938	123.11	Passed	Passed	0.0213	0.0380
22	Nonioside A *	10179753	410.4	Failed	Passed	0.2690	0.0835
23	Nonioside D *	10741757	440.4	Failed	Passed	0.0610	0.2024
24	Nonioside E *	44423082	602.6	Failed	Passed	0.0471	0.2919
25	Pantothenic acid	6613	219.23	Passed	Passed	0.0006	0.0632
26	Phenylalanine	6140	165.19	Passed	Passed	0.0330	0.0001
27	Proline	145742	115.13	Passed	Passed	0.1314	0.0535
28	Pyridoxine	1054	169.18	Passed	Passed	0.0302	0.0751
29	Riboflavin	493570	376.4	Passed	Passed	0.0012	0.0214
30	Tyrosine	6057	181.19	Passed	Passed	0.0011	0.0017
31	Vanillic acid	8468	168.15	Passed	Passed	0.0022	0.1557

* Conditional acceptance. Abbreviations: CID: PubChem Compound ID; MW: molecular weight, BBB: blood–brain barrier; F20%: human oral bioavailability.

**Table 2:** Vina docking scores of *Morinda citrifolia* bioactive compounds with GRIA2. Common interacting residues between perampanel and the compounds are highlighted in bold.

Compound	Vina Score (kcal/mol)	Binding Site ^#^ (PDB ID: 7F3O)
Glutamic Acid (PubChem ID: 33032)	−6.6	**ILE421**, **LEU422**, **GLU423**, **SER424**, **VAL427**, **MET428**, **GLY469**, LYS470, TYR471, PRO499, LEU500, THR501, ARG506, LEU671, SER673, GLY674, SER675, THR676, LYS677, **ARG705**, **THR706**, **THR707**, **ALA708**, **GLU709**, **VAL711**, TYR723, LEU724, LEU725, GLU726, MET729, **TYR732**, **ILE733**, TYR753
Kaempferide (PubChem ID: 5281666)	−5.6	**ILE421**, **LEU422**, **GLU423**, **SER424**, **VAL427**, **MET428**, **MET429**, **ILE465**, **VAL466**, **GLY467**, **ASP468**, **GLY469**, LYS470, **ARG705**, **THR706**, **THR707**, **ALA708**, **GLU709**, **VAL711**, **ALA712**, **ARG715**, **TYR732**, **ILE733**, **ARG736**, **LYS737**, PRO738, **GLU793**
Riboflavin (PubChem ID: 493570)	−6.5	**ILE421**, **LEU422**, **GLU423**, **SER424**, **VAL427**, **MET428**, **MET429**, **ILE465**, **VAL466**, **GLY467**, **ASP468**, **GLY469**, TYR471, LEU671, **ARG705**, **THR706**, **THR707**, **ALA708**, **GLU709**, **VAL711**, **ALA712**, **ARG715**, **TYR732**, **ILE733**, **ARG736**, **LYS737**, PRO738, **GLU793**
Perampanel * (PubChem ID: 9924495)	−6.1	ILE421, LEU422, GLU423, SER424, VAL427, MET428, MET429, ILE465, VAL466, GLY467, ASP468, GLY469, ARG705 THR706, THR707, ALA708, GLU709, VAL711, ALA712, ARG715, TYR732, ILE733, ARG736, LYS737, GLU793

* Control; ^#^ binding site details were taken from CbDock2.

**Table 3:** Vina docking scores of Nonioside E with PRKCA. Common interacting residues between nonioside E and the controls are highlighted in bold.

	VINA Score (kcal/mol)	Binding Site ^#^ (PDB ID: 4DNL)
Nonioside E (PubChem ID: 44423082)	−7	THR251, ASP254, ASP248, PHE255, ASN279, GLN280, ARG159, ASP246, ASP193, SER192, PRO194, ILE244, LEU277, LEU183, ILE184, GLY257, PRO185, ASP187, ARG252
Staurosporine * (PubChem ID: 44259)	−6.6	**THR251**, THR250, ASN189, SER217, ARG216, **ASP193**, TRP247, ASP187, **ASP248**, ARG249, PRO188
14(R)-Hydroxy-retro-vitaminA * (PubChem ID: 6438154)	−6.8	**LEU277**, **GLY257**, **ARG159**, **ILE184**, **PRO185**, **ASP254**, **THR251**, **ASP248**, PRO188, **ASP187**, **SER192**, **PHE255**, **ILE244**, **ASP246**, TRP245, **PRO194**, **LEU183**

* Control; ^#^ binding site details were taken from Discovery Studio Visualizer.

## Data Availability

The data and results presented in this study are available upon request from the authors.
